# Age Differences in Positive Event Appraisals during COVID-19: Evidence from a Daily Diary Study

**DOI:** 10.1093/geroni/igab046.3208

**Published:** 2021-12-17

**Authors:** Patrick Klaiber, Lydia Ong, Anita DeLongis, Nancy Sin

**Affiliations:** University of British Columbia, Vancouver, British Columbia, Canada

## Abstract

Multiple studies suggest that community-dwelling older adults are psychologically resilient in the face of the COVID-19 pandemic. Notably, during the initial weeks of the COVID-19 outbreak, older age was associated with engaging in more daily positive events (Klaiber et al., 2021, Journal of Gerontology: Psychological Sciences). We followed up on these findings by exploring age differences in positive event appraisals during the COVID-19 pandemic. During the 7-day diary study conducted between March and August 2020, 1036 participants (mean age = 45.95, SD = 16.04, range = 18-91) reported their positive events in nightly surveys. If at least one positive event occurred, participants rated their appraisals of the event on the following dimensions: importance, calmness, happiness, gratitude, personal responsibility, and control. Older adults (60 years+) rated their positive events to be more personally important and felt more calm and happy during these events, compared to younger (18-39 years) and middle-aged adults (40-59 years). Furthermore, older adults felt more grateful during positive events compared to younger but not middle-aged adults. There were no age differences in feelings of control or personal responsibility for positive events. These findings highlight the importance of daily positive events for older adults during a time of major stress. In line with theories on adult development, daily positive event processes in older adults are characterized by valuing positive and meaningful social connections, as well as a greater degree of positive event-specific emotions such as feeling calm, happy, and grateful.

